# De novo prediction of DNA-binding specificities for Cys_2_His_2_ zinc finger proteins

**DOI:** 10.1093/nar/gkt890

**Published:** 2013-10-03

**Authors:** Anton V. Persikov, Mona Singh

**Affiliations:** ^1^Lewis-Sigler Institute for Integrative Genomics, Princeton University, Princeton NJ 08544, USA and ^2^Department of Computer Science, Princeton University, Princeton NJ 08544, USA

## Abstract

Proteins with sequence-specific DNA binding function are important for a wide range of biological activities. *De novo* prediction of their DNA-binding specificities from sequence alone would be a great aid in inferring cellular networks. Here we introduce a method for predicting DNA-binding specificities for Cys_2_His_2_ zinc fingers (C2H2-ZFs), the largest family of DNA-binding proteins in metazoans. We develop a general approach, based on empirical calculations of pairwise amino acid–nucleotide interaction energies, for predicting position weight matrices (PWMs) representing DNA-binding specificities for C2H2-ZF proteins. We predict DNA-binding specificities on a per-finger basis and merge predictions for C2H2-ZF domains that are arrayed within sequences. We test our approach on a diverse set of natural C2H2-ZF proteins with known binding specificities and demonstrate that for >85% of the proteins, their predicted PWMs are accurate in 50% of their nucleotide positions. For proteins with several zinc finger isoforms, we show via case studies that this level of accuracy enables us to match isoforms with their known DNA-binding specificities. A web server for predicting a PWM given a protein containing C2H2-ZF domains is available online at http://zf.princeton.edu and can be used to aid in protein engineering applications and in genome-wide searches for transcription factor targets.

## INTRODUCTION

The ability of proteins to recognize and bind specific DNA regions is critical in a range of key biological processes, including transcription, replication, packaging, repair and recombination. Sequence-specific DNA recognition by transcription factors is of particular interest due to its role in dictating when and where proteins are expressed. Despite recent progress in experimentally mapping protein–DNA \interactions (1,2), there is no organism for which a near-complete regulatory network is known: high-throughput experiments are still imperfect and time-consuming, and it is not feasible in the near future to apply them in all conditions and/or genomes of interest. Thus, reliable computational methods for quick but accurate prediction of protein–DNA interactions are necessary to help fill this gap (3,4).

Cys_2_His_2_ zinc finger (C2H2-ZF) proteins represent the largest class of DNA-binding proteins in metazoans. C2H2-ZF proteins have been implicated in several developmental, cell proliferation and complex disease pathways (5). Structural studies have revealed a highly conserved DNA-binding interface (6), with a proposed ‘canonical’ model suggesting that DNA-binding specificity is due to four amino acid-nucleotide contacts per C2H2-ZF domain. C2H2-ZF proteins have been intensely studied, with thousands of experimentally determined examples of protein–DNA pairs, largely based on the Zif268 model system, that are known to either bind or not. Nevertheless, the binding specificities of most C2H2-ZFs within genomes are not known: for example, in the human genome, of the ∼675 proteins annotated with C2H2-ZF domains (7), specificities have been determined for less than a hundred (8).

Several computational approaches have been developed to infer statistical pairwise contact energies between amino acids in C2H2-ZF domains and the corresponding nucleotides of DNA (9–14). We have previously shown that inferring these contact energies via support vector machines (SVMs) yields accurate predictions of whether a C2H2-ZF protein can bind a specific DNA site and outperforms previously described approaches (12). Our SVM-based approach has also been shown by others to be effective in predicting positional base pair preferences of yeast C2H2-ZF proteins (15), and has been used to help implicate the C2H2-ZF protein PRDM9 in meiotic recombination by matching a motif associated with recombination hotspots with predictions based on our approach (16). We have also since performed additional statistical and structural analysis of C2H2-ZF protein–DNA interfaces and have uncovered empirical evidence suggesting that contacts beyond the canonical ones that we previously used in our models are important for and improve predictions of C2H2-ZF protein–DNA interactions (17); this is consistent with the previously noted limitations of the canonical binding model (6).

Here we combine our findings on an expanded structural interface for C2H2-ZF–DNA binding with our approach for inferring pairwise statistical contact energies to develop a method for accurate *de novo* prediction of DNA-binding specificities from protein sequences alone. We describe how to leverage approaches that compute empirical energy scores of binding interfaces in order to build position weight matrices (PWMs) representing binding specificities for C2H2-ZF domains. We predict DNA-binding specificities on a per-finger basis and then merge predictions for C2H2-ZF domains that are arrayed within sequences. The described approach is implemented as a front-to-end online webform (http://zf.princeton.edu) that first uncovers C2H2-ZF domains and then predicts a PWM describing the binding specificities of these domains. In order to benchmark our approach, we gather a diverse data set of 143 non-redundant naturally occurring C2H2-ZF proteins with known specificities. We note that to date most approaches for predicting C2H2-ZF binding sites have been tested either on artificial zinc fingers where the binding interface is known or on natural proteins limited to a few (e.g. at most 4) canonically linked C2H2-ZF domains (10–13); in contrast, our test set contains proteins with a diverse set of architectures that may contain numerous C2H2-ZF domains, only some of which are responsible for the reported binding specificities. We present an evaluation framework based on PWM alignment and demonstrate good agreement between our predicted PWMs and those known from experiment. Further, we show via case studies that our predicted PWMs can be used with alignments to experimental PWMs to identify which fingers or protein variants are mediating the observed DNA-binding specificities.

## MATERIALS AND METHODS

### Structural model and prediction algorithms

**Figure 1.**
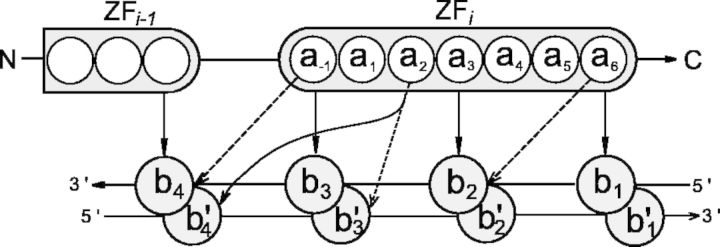
Schematic representation of the C2H2-ZF protein–DNA interaction interface, with 2 successive fingers shown. Amino acids within the *i-*th finger are numbered according to their relative position from the start of the alpha helical domain, with a_−1_ denoting the residue immediately preceding the helix. Bases b_1_, b_2_, b_3_ and b_4_ are numbered sequentially from 5′ to 3′ of the primary DNA strand, and the complementary bases are primed. The canonical contacts are shown with solid arrows, and the three additional contacts in the expanded binding model are shown with dashed arrows.

Structural studies have revealed that each ‘finger’ in a C2H2-ZF protein consists of a ββα structure, with four amino acids in the α-helix (referred to as positions -1, 2, 3 and 6) largely determining DNA-binding specificity (6). Each finger binds a 4-bp region and previous structural analysis has suggested a canonical binding model with four contacts: *a_6_b_1_, a_3_b_2_, a_-1_b_3_* and *a_2_b_4_* (Figure 1). However, we have recently found that up to three additional contacts may be important for C2H2-ZF binding specificity: *a_2_b_3_*, *a_-1_b_4_* and *a_6_b_2_*, with the importance of contact *a_2_b_3_* supported by several lines of evidence (17). Thus, in the current study, two structural models are considered and tested: the canonical binding model assuming four amino acid–nucleotide contacts and the expanded binding model with seven contacts (the canonical model plus the *a_2_b_3_*, *a_-1_b_4_* and *a_6_b_2_* contacts). We also include a second degree polynomial kernel based on the canonical model, which implicitly takes higher order interactions into account (12,17).

We previously built a literature-derived experimental database including 1312 positive and 8081 negative examples of C2H2-ZF protein–DNA binding (12). Assuming the binding models described above, every protein–DNA interface can be represented in a per-finger manner as a vector ***x*** with dimensionality either 320 (for the canonical binding model: 4 × 4 × 20) or 560 (for the expanded binding model: 7 × 4 × 20), where ***x****_abc_* = 1 if amino acid *a* and base *b* comprise contact *c* in the finger. Three SVMs are trained using this data set: a linear and a second-degree polynomial SVM trained on the basis of the canonical binding model and a linear SVM trained on the basis of the expanded binding model. We refer to these methods as the ‘canonical’, ‘polynomial’ and ‘expanded’ SVMs. SVMs are trained using SVM-light version 6.02 (18), with the constraint that the weight vector go through the origin. The regularization parameter is chosen automatically by SVM-light. We note that with linear SVMs, the *abc*-th dimension of the trained weight vector ***w*** represents the empirical contact energy between amino acid *a* and base *b* when they comprise contact *c*, and the score for a particular C2H2-ZF protein–DNA configuration represented as ***x*** is computed as ***w*·*x***.

### Predicting position weight matrices for zinc finger domains

Given any approach for empirically scoring C2H2-ZF protein–DNA interfaces, we will use it to predict a PWM for a specific C2H2-ZF protein. We note that in the formulas that follow, for notational simplicity, we assume a fixed C2H2-ZF domain and do not explicitly parameterize on the domain. We begin by assuming that each C2H2-ZF domain binds independently to a corresponding 4-bp DNA site. There are 256 sequence combinations of such 4 bp binding sites. Therefore, for a given C2H2-ZF domain, using any of the scoring methods described above, we can compute an empirical binding energy *E_i_* for each of the 256 possible 4 bp sequences. In other words, every potential 4 bp binding site can be considered as the realization of a statistical-mechanical system of 256 independent states. The probability that a given C2H2-ZF domain occupies any of these states *i* (i.e. binds the corresponding 4 bp sequence) is assumed to follow the classical Boltzmann distribution as previously suggested (4,19):
(1)
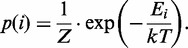



For the specific case when a trained SVM is used for evaluating a protein’s ability to bind a 4-bp sequence *i*, we assume that the SVM score *y_i_* (e.g. *y_i_* = ***w*·*x_i_*** in the linear kernel case, where ***w*** is the weight vector, and ***x_i_*** is the vector representing the protein–DNA interface being considered) is negatively proportional to the binding energy *E_i_*. Therefore, the probability that the C2H2-ZF domain binds a certain 4 bp sequence *i* is given by:
(2)
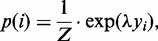

where λ is the constant factor that converts SVM scores into units of *kT* and Z is the partition function (computed as a normalization parameter so that a probability distribution is obtained). For the results reported here, we use λ = 4 for all three methods; variations in λ affect the information content of the predicted PWMs but do not otherwise greatly affect our overall findings.

The probability of every given position *pos* in the 4-bp DNA sequence to be occupied by a nucleotide *b* can then be determined from a summation of probabilities for 4 bp sequences with nucleotide *b* in position *pos*:
(3)
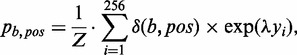

where *b* ∈ {A,C,G,T} and *pos* ∈ {1,2,3,4}, and *δ(b, pos)* = 1 if site *i* has nucleotide *b* in position *pos*. For simplicity, we assume that nucleotides are chosen with equal *a priori* probabilities (i.e. all four nucleotides are equally common in the genome). We note that to address situations with strongly biased genomes (e.g. in *Plasmodium falciparum* (20)), background nucleotide distributions could be incorporated into the formula. Once per-position nucleotide distributions are predicted, sequence logos are generated as described elsewhere (21) using WebLego (22).

**Figure 2.**
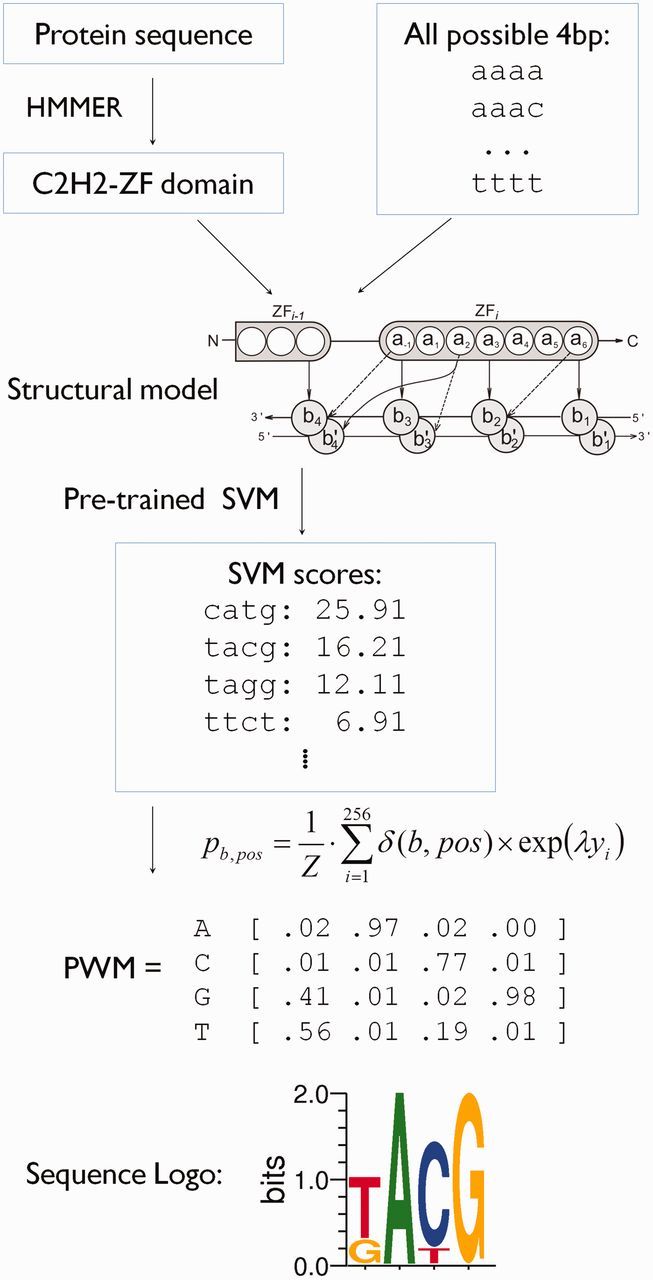
Flow chart for per-finger prediction of PWMs. The ability of every individual zinc finger domain (as predicted by HMMER (32)) to bind all possible 4 bp DNA sequences is scored; here we show a pre-trained SVM that used the seven-contact model. The SVM scores for all possible 256 DNA variants are subsequently used to compute the final PWM using equation (3). Finally, the PWM is visualized in the form of a sequence logo.

Importantly, this method takes advantage of the fact that SVM scores can be obtained for all 256 4-bp sequences. This is in contrast to traditional methods for building PWMs from experimental data, where a potentially small number of binding sites are uncovered and used. Our approach instead uses all sites to predict positional nucleotide probabilities without applying an arbitrary cutoff for binding sites. An overview of the procedure for predicting per-finger PWMs is shown in Figure 2.

We note that for the linear SVMs, it is not necessary to enumerate over all possible 4 bp sequences, and instead the SVM scores for each nucleotide position can be considered independently.

### Combining per-finger PWMs for adjacent C2H2-ZF domains

The above approach predicts a 4-bp PWM for a single C2H2-ZF domain independent of any other domain within the protein sequence. However, successive C2H2-ZF domains that bind DNA together typically do so in overlapping subsites of length 4 (Figure 1). In the canonical model, two amino acids affect the overlapping nucleotide position ***b_4_***: residue ***a_6_*** from the preceding ZF*_i-1_* domain interacts with the primary DNA chain and residue ***a_2_*** of the current ZF*_i_* domain interacts with the complementary strand nucleotide ***b_4_′*** (Figure 1). Thus, for arrayed C2H2-ZF domains, predicted per-finger PWMs need to be ‘merged’ to account for this overlap. Further, it may be desirable to weigh the contribution of each of the contacts differently when computing the merged PWM. Therefore, to predict the probability distribution for such a ‘junction nucleotide’ position, we introduce a parameter 0 ≤ *α* ≤ 1 so that the overlapping nucleotide probabilities *p_b_* can be predicted from the probabilities *p_b__,__i-1_* and *p_b__,__i_* computed independently for the same nucleotide position using the corresponding ZF*_i-1_* and ZF*_i_* domains as:
(4)
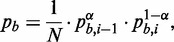

where *N* is a normalization factor that ensures a proper probability distribution at the overlapping nucleotide. Note that whereas *α* = *0* ignores the effect of residue ***a_6_*** in the ZF*_i-1_* domain and *α* = 1 ignores the contribution of residue ***a_2_*** in the ZF*_i_* domain, *α* = 0.5 results in an equal weighting of either domain with equation (4) computing the geometric means of the probabilities. For the results described in this article, we use *α* = 0.75 to merge per-domain PWMs; this parameter is chosen to reflect the closer proximity of residue ***a_6_*** to the primary DNA chain and therefore its likely higher contribution to DNA binding. However, we note that using *α* = 0.5 does not significantly alter our findings.

### Arrays of C2H2-ZF domains

The total number of C2H2-ZF domains in a single protein can vary immensely; for example, the human OAZ protein (NCBI accession no: Q2M1K9) has 30 C2H2-ZF domains. However, consecutive C2H2-ZF domains are thought to ‘canonically’ bind the major groove of DNA only when connected by short linkers (6). Further, in several studied cases, DNA-binding C2H2-ZF domains that are far away from each other in sequence appear to bind DNA independently (23). Therefore, within each protein sequence, we array C2H2-ZFs together based on their proximity along the protein sequence, and predict specificities at the per-array level. Specifically, based upon the analysis of known co-crystal structures of C2H2-ZF–DNA complexes, we hypothesize that C2H2-ZF domains can act together as a single DNA-binding array if they have a sequential distance between them from 9 to 12 amino acids (as counted from the last Histidine and the first Cysteine in the following C2H2-ZF domain). Therefore, we array C2H2-ZF domains together if there are at most 12 residues between them, while those separated by longer linkers are considered as separate arrays. In our analysis, the total number of C2H2-ZF domains in a single array has no maximum limit. For example, the human CTCF protein (P49711) has an array of 11 C2H2-ZF domains. For each array, PWMs are predicted per-domain and then merged within the array using the strategy outlined above. If an array has *i* zinc fingers, then the length of the predicted PWM is *3i+1*.

### Experimental databases

We gather C2H2-ZF protein specificities obtained from four resources (described below) and consider performance on the combined data set of all proteins, as well as on the individual data sets. For each individual data set, and then for the combined data set, PWMs for protein sequences that are identical in amino acid positions from −1 to 6 in each of their ZF domains are considered together. Further, each experimental PWM in these databases is trimmed to remove low information content columns at the ends. Specifically, for each column *m* in a PWM, we compute the information content (*IC*) as:
(5)
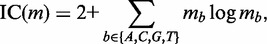

where *m_b_* represents the frequency of the base *b* in column *m* (24). We trim columns with IC < 0.5 from both the 5′ and 3′ sides.

#### JASPAR database

The JASPAR database (http://jaspar.genereg.net) contains high-quality data for transcription factor specificities in the form of PWMs (25). C2H2-ZF proteins are found in JASPAR under the class ‘Beta-Beta-Alpha-zinc finger, Zinc-coordinating’ and 66 proteins contain at least one array of two or more C2H2-ZF domains. Once we group proteins that are identical in their −1 to 6 C2H2-ZF positions, we have 57 proteins.

#### UniProbe database

The UniPROBE (Universal PBM Resource for Oligonucleotide Binding Evaluation) database (26) includes PWMs determined via the protein binding microarray (PBM) technology (27). PWMs for 26 C2H2-ZF mouse proteins (28) and 14 yeast proteins (29), as determined by the authors via the Seed-and-Wobble algorithm (30), are available from http://thebrain.bwh.harvard.edu/uniprobe/. These yield 23 mouse and 13 yeast proteins that are non-redundant. We note that this database contains two additional yeast C2H2-ZF proteins (SFP1 and STP2) but their C2H2-ZF domains are not arrayed so they are not considered here.

#### Jolma database of human transcription factors

Recently, Jolma *et al.* determined 830 DNA-binding profiles for various human transcription factors via high-throughput SELEX and ChIP sequencing (8). In this data set, 57 PWMs are reported for 52 clones of 46 human C2H2-ZF proteins (8). After grouping redundant proteins, we obtain 36 C2H2-ZF proteins in this data set.

#### FlyFactorSurvey database

The FlyFactorSurvey database contains PWMs for transcription factors in *Drosophila melanogaster*, as determined largely by the bacterial one-hybrid system (31). This database includes 118 motifs for 55 C2H2-ZF proteins. Experimental PWMs and the specific protein fragments used in the experimental screens are available from http://pgfe.umassmed.edu/TFDBS/. We compare the specified protein fragments and obtain 52 non-redundant proteins.

#### Combined data set

After merging redundant protein sequences, the combined data set contains 143 proteins. In total, the proteins in this combined data set contain ∼1400 columns in their trimmed PWMs.

### Evaluation framework

In order to test our procedure for predicting PWMs, for each protein we compare our predicted PWMs for these proteins to those that have been experimentally reported. We note that the transcription factors in the test set are independent of those in the experimental data set used to train the SVMs, as the training data set consists of examples derived largely from synthetic C2H2-ZF proteins based on the Zif268 system (12). Further, whereas it is known which C2H2-ZF domains in our training set interact with specific DNA subsites, the test set consists of PWMs corresponding to DNA-binding specificities at the protein level; that is, these resources do not specify which C2H2-ZF domains within the protein are responsible for the reported specificities.

For each transcription factor in our test sets, we uncover C2H2-ZF domains using HMMER (32), as described previously (17). We consider only proteins that contain at least one array of two or more C2H2-ZF domains. PWM predictions are made for all discovered arrays of size at least two and these PWMs are compared to the experimentally determined DNA-binding specificities. Every C2H2-ZF array is evaluated independently and a prediction is assumed to be correct for every protein where at least one C2H2-ZF array matches the known PWM, as determined by a PWM alignment algorithm (see below). When multiple experimental PWMs are available for a protein in a database, the prediction is assumed to be correct if a good match is observed for at least one experimental PWM.

### PWM alignments

We align a predicted PWM with an experimental PWM using a column score based on the Pearson correlation coefficient (PCC), but which down weights the contributions of columns in the experimental PWM that have low information content. In particular, in the IC-weighted PCC measure, the similarity of predicted column *n* and experimentally determined column *m* where *n_b_* and *m_b_* give their nucleotide frequencies is computed as:
(6)
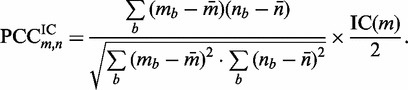

The maximum scoring local alignment using this measure between the predicted PWM and the experimental PWM is determined, where the reverse complement of the experimental matrix is also considered. Gaps within the alignment are not allowed. We note that we have also considered several other column measures for PWM alignment including Jensen–Shannon Divergence, Kullback–Leibler Divergence, Pearson Correlation Coefficient, Euclidean Distance (33–36), and average log likelihood ratio (37), and the overall conclusions are consistent with what we report here; however, in other applications, depending upon the overall quality and/or the IC of the predictions, it may be better to use a measure other than IC-weighted PCC or to use the ICs of both the predicted and actual PWMs in computing column scores.

Once the best alignment of the predicted and experimental PWM is obtained, we use several approaches to assess performance. In the first approach, we consider the range of IC-weighted column scores obtained in the alignments for each column in a trimmed experimental PWM. In the second approach, we classify each column in the experimental PWM as correctly or incorrectly predicted, and compute the fraction of columns that are correctly predicted. Specifically, we judge an experimental column to be correctly predicted if the IC-weighted PCC between it and the predicted column aligned to it is at least 0.25. (See Supplementary Figures S1 and S2 for ranges and examples of IC-weighted PCC scores.) In the final approach, prediction accuracy is evaluated by computing empirical *P*-values, where the final alignment score using the IC-weighted PCC measure is compared with the distribution of scores calculated for 10 000 randomized matrices of the size of the original experimental PWM. Randomized matrices are generated by picking random columns from PWMs in the Jaspar database; only PWMs from the organism corresponding to the experimental matrix (*fungi, drosophila or vertebrata*) are used. A prediction is considered to be correct for cases with *P* ≤ 0.05 for at least one C2H2-ZF array and any experimental PWM for the protein under consideration.

## RESULTS

### Quality of PWM predictions for natural PWMs

**Figure 3.**
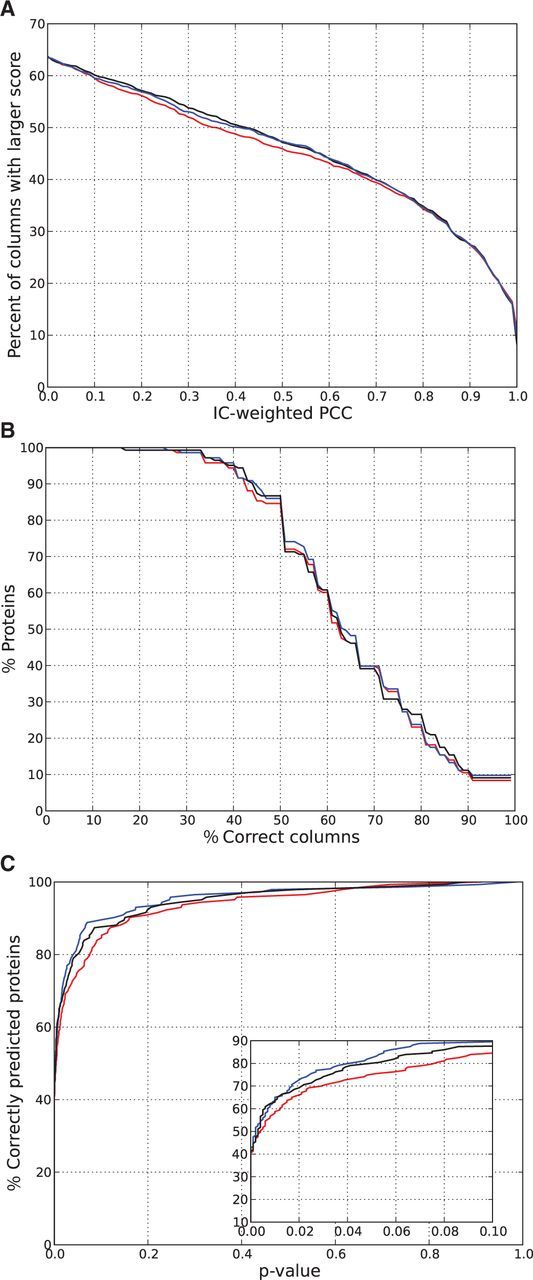
Performance of canonical (red), expanded (blue) and polynomial (black) SVMs in predicting natural C2H2-ZF proteins in the combined data set. (**A**) Distribution of IC-corrected PCC scores in aligned PWMs. For each threshold of IC-corrected PCC score (*x*-axis), we plot the fraction of columns in trimmed experimental PWMs that achieve a score that high in their alignments to predicted PWMs. (**B**) Fractions of correctly predicted positions in PWMs. We give the percent of proteins whose PWMs (*y*-axis) have at least a given percent of correct columns (*x*-axis), using an IC-corrected PCC threshold of 0.25 to determine whether an aligned column is correct. (**C**) The percent of correctly predicted proteins at various *P*-values.

For each protein in our combined database, we predict a PWM for each array and then determine the best alignment of an array for a protein with the experimentally determined PWM. We first evaluate how well the columns in our combined test set are predicted (Figure 3A). Our combined test set contains ∼1400 columns in their PWMs, and we find that ∼55% of the columns in our data set have IC-weighted PCC scores greater than or equal to 0.25 using either the canonical, expanded or polynomial SVMs. In fact, while a range of column score are observed, 63.5% of the columns in experimental PWMs (using either canonical, expanded or polynomial SVMs) are in at least weak agreement with their predictions as judged by having an IC-weighted PCC scores greater than or equal to 0.

We next evaluate how well individual PWMs are predicted. For each aligned PWM, we compute the fraction of its columns that are correct. For this analysis, we consider a column to be correct if it has an IC-weighted PCC score >0.25, and consider what fraction of proteins have an increasing fraction of the columns in their PWMs correctly predicted (Figure 3B). For all three approaches, >85% of proteins have 50% of the columns in their PWMs correctly predicted, and >30% of proteins have 75% of their columns correctly predicted. Further, using any of the three methods, half the proteins have at least 60% of their PWM columns correct, and three quarters have >50% of their columns correct. We note that low IC columns (IC < 0.5) cannot achieve an IC-weighted PCC score >0.25 (Supplementary Figure S2), and thus this approach may give a pessimistic view of performance.

As an alternate approach to evaluate predicted PWMs, we consider for each of the three methods the empirical *P*-value of obtaining a score at least as high as that obtained by aligning the experimental and predicted PWMs (see ‘Materials and Methods’ section). Then, as we vary the *P*-value threshold, we consider the fraction of proteins with experimental PWMs whose alignments with the predicted PWM are significant at that level (Figure 3C). The expanded and polynomial SVMs outperform the canonical SVM, as an increased number of proteins are considered correctly predicted at a wide range of *P*-values. At *P* ≤ 0.05, the expanded SVM model has statistically significant matches to 83% of the proteins in the combined data set, while the canonical and polynomial SVMs have matches to 75 and 80% respectively.

For each of the individual test sets, we give the fraction of PWMs correctly predicted at *P* ≤ 0.05 (Table 1). We also give, for each data set, the full *P*-value curves, the distribution of column scores and the distribution of the fraction of correctly predicted experimental PWM positions in Supplementary Figures S3–S7. All SVM methods exhibit the highest performance on the yeast data set, with the expanded linear SVM obtaining 12 correctly predicted proteins (92%) at *P* ≤ 0.05. Over 87% of fly transcription factors are predicted at *P* ≤ 0.05 by all SVM methods (Table 1). The best overall performance is shown by the polynomial and expanded linear SVMs, with 90% of the fly proteins correctly predicted. We note that all the approaches perform significantly better for yeast and fly proteins than for proteins in other species. In contrast, the methods have lowest performance on the mouse PBM data set, where only 61% of the proteins are predicted correctly by the expanded SVM. Performance on the JASPAR and Jolma human data sets are intermediary, with 75 and 81% respectively of the proteins correctly predicted at *P* ≤ 0.05 using the expanded linear SVM. The differing performances of the methods on these data sets is most likely due to the varying complexities of the domain architectures found in each of them. For example, the yeast data set consists entirely of proteins with two C2H2-ZF domains whereas the mouse data set has the highest fraction (46%) of proteins with five or more C2H2-ZF domains.

**Table 1.** Number and fraction (in parentheses) of proteins in each data set for which predicted PWMs significantly match at *P* ≤ 0.05

Database	Number of proteins	SVM models		
		Linear canonical (%)	Linear expanded (%)	Polynomial (%)
JASPAR	57	37 (65)	43 (75)	41 (72)
UniProbe yeast	13	10 (77)	12 (92)	10 (77)
UniProbe mouse	23	14 (61)	14 (61)	16 (70)
FlyFactorSurvey	52	45 (87)	47 (90)	47 (90)
Human	36	25 (69)	29 (81)	28 (78)
Combined	143	107 (75)	118 (83)	114 (80)

The best performing method on each data set is shaded.

Using all three evaluation measures, the expanded SVM outperforms the canonical SVM and in many cases the polynomial SVM. A larger fraction of experimental PWM columns have IC-weighted PCC score >0.25 using the expanded and polynomial SVMs than the canonical SVM (Figure 3A). For a wide range of thresholds, a larger fraction of PWMs using the expanded SVM have that threshold of columns correct as compared to the canonical SVM (Figure 3B). Further, a larger fraction proteins in the combined data set have statistically significant matches to the PWMs predicted using the expanded SVM than predicted using the canonical SVM (Figure 3C) or a previously published method (11) based on the canonical binding model (Supplementary Figure S8). The expanded SVM also outperforms the canonical SVM on all the individual data sets except the mouse PBM data (Table 1). Overall, the performance of the expanded SVM supports the importance of the three additional contacts included in the expanded seven-contact model that are not part of the canonical model (17).

### Factors affecting the quality of PWM predictions

**Figure 4.**
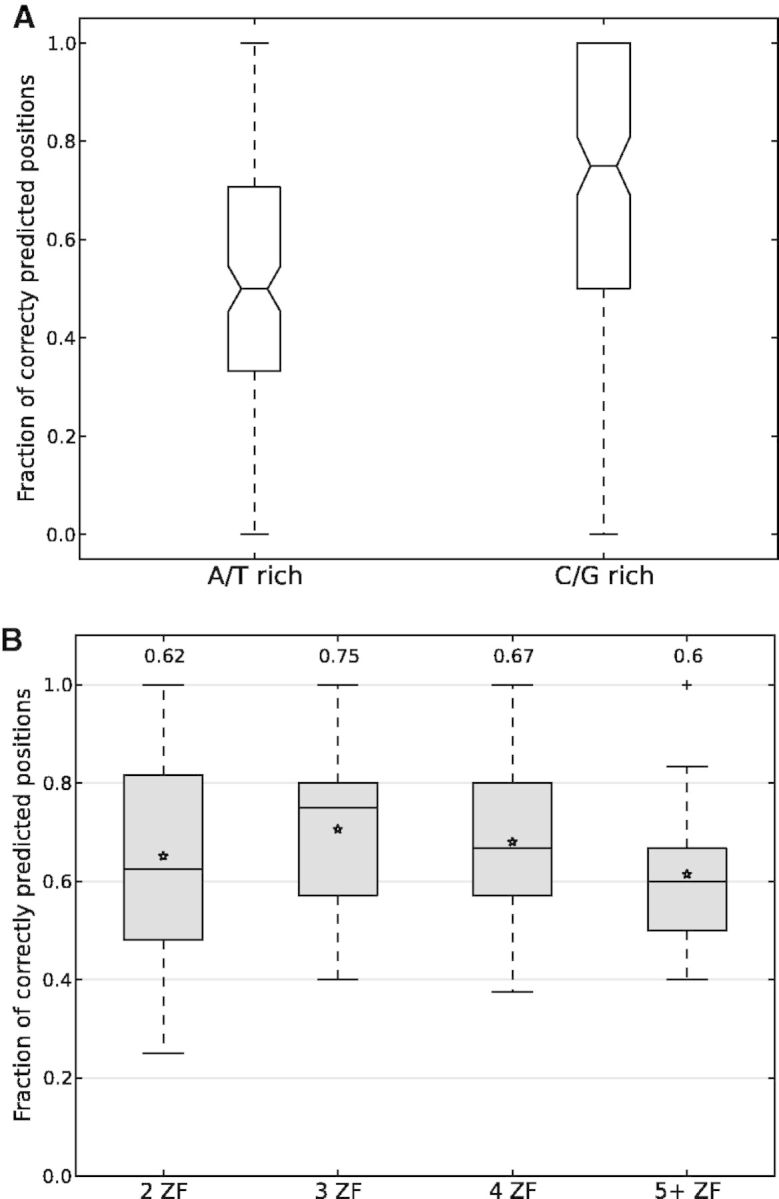
(**A**) The fraction of correctly predicted PWM positions based upon nucleotide composition. For each protein in the combined data set, we compute the fraction of its A/T-rich and C/G-rich columns that are correctly predicted by the expanded linear SVM and display this data with a box plot. Significantly better performance is observed for positions with high C/G content than those with high A/T content (*P* < 10^−10^ using the Mann–Whitney test). (**B**) The fraction of correctly predicted PWM positions based upon the number of C2H2-ZF domains. For each protein in the combined data set, we classify it based upon the number of arrayed C2H2-ZF domains yielding the best alignment with the experimental PWM using the expanded SVM. For each classification (2, 3, 4 or 5+ C2H2-ZF domains), we use a box plot to display the fraction of correctly predicted positions per protein, and give the median value above each box. Performance is highest for arrays with three C2H2-ZF domains and declines as the number of domains increases. For both (A) and (B), the bottom and top of the box plots are the 25th and 75th percentiles (i.e. they give the inter-quartile range), and whiskers on the top and bottom represent the maximum and minimum data points within the range represented by 1.5 times the inter-quartile range. Further, in both (A) and (B), positions are judged to be correct using an IC-weighted PCC score of at least 0.25.

We have previously shown that our training data set has more examples with C/G nucleotides relative to A/T nucleotides, which are most likely underrepresented (17). This may lead data-driven methods to not recognize amino acid positional preferences for A and/or T bases. In order to test the performance of our methods in predicting PWM columns with different nucleotide preferences, we focus on two subgroups of columns in our experimental PWMs: those whose combined fraction of C and G nucleotides is >0.75 (‘C/G-rich’) and those whose combined fraction of A and T nucleotides is >0.75 (‘A/T-rich’). For each protein in the combined data set, we compute the fraction of its A/T-rich and C/G-rich columns in its aligned experimental PWM that are correctly predicted by the expanded linear SVM, as judged by an IC-weighted PCC score of at least 0.25 (Figure 4A). Across the proteins, we observe significantly better performance in predicting positions with high C/G content than those with high A/T content (median 75% versus 50%, *P* < 10^−^^10^ using the Mann–Whitney test). Indeed, over the entire data set, 73% of the C/G-rich positions are correctly predicted by the expanded SVM, whereas this number is only 50% for the A/T-rich positions. The canonical and polynomial SVMs also show a similar gap in performance in predicting A/T-rich positions (data not shown).

We also assess the performance of our methods in predicting C2H2-ZF binding specificities based upon the number of C2H2-ZF domains within arrays. Using the expanded SVM, for each protein in our combined data set, we consider the best alignment between the predicted and experimental PWMs and determine the number of C2H2-ZF domains in the array used to make the prediction. We then classify each protein based on this number, and assess what fraction of its experimental PWM columns are correctly predicted (Figure 4B). Performance is highest for arrays with three domains, and decreases with an increasing number of C2H2-ZF domains; this is also true for the canonical and polynomial SVMs (data not shown). The apparent lower performance on arrays with two C2H2-ZF domains, as compared to three and four domain arrays, is most likely due to the length distribution of experimental PWMs. By visual inspection, we observe that many of the experimental PWMs for proteins with two C2H2-ZF domains have low IC columns at the 5′ and 3′ ends even after trimming. Indeed, theoretically, C2H2-ZF domains that bind in a canonical manner would have 7, 10, 13 and 16 bases in their PWMs for 2, 3, 4 and 5 domains, respectively; however, experimental PWMs have on average 9.3, 9.2, 10.2 and 10.8 bases respectively for these cases. Based on this analysis, we conclude that, as expected, specificity is easiest to predict for proteins with a smaller number of C2H2-ZF domains.

### Description of webpage for predicting PWMs for C2H2-ZF proteins

We have implemented the described approach for predicting DNA-binding specificities for C2H2-ZF proteins and have made it available at http://zf.princeton.edu. Our webserver allows users to input protein sequences, and C2H2-ZF domains are identified via HMMER (32). Given the difficulties in predicting DNA-binding specificities for proteins with multiple C2H2-ZF domains, we allow the user to specify which domains comprise the array for which predictions should be made and which method (expanded or polynomial) should be used to predict the PWM. Finally, the predicted PWM is displayed as a sequence logo and can be downloaded in matrix form.

### Case studies: uncovering C2H2-ZF domains mediating specificity via PWM alignment

A single C2H2-ZF gene may encode numerous proteins with differing DNA-binding specificities through the use of alternative splicing, where different C2H2-ZF domains are included in various isoforms. Here we show that it is possible to distinguish isoforms based upon their predicted DNA-binding specificities by focusing on two *D. melanogaster* proteins with more than one isoform with known specificity: tramtrack and lola.

**Figure 5.**
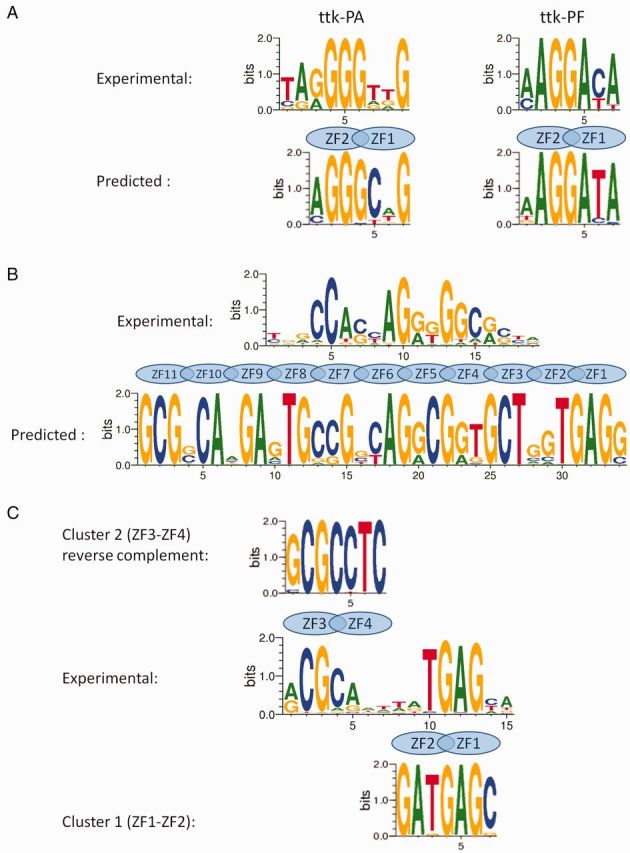
Comparison of experimental and SVM predicted sequence logos for (**A**) Tramtrack: experimental (top) and predicted by polynomial SVM (bottom); (**B**) CTCF: experimental (top) and predicted by expanded SVM (bottom); and (**C**) HER: predicted by expanded SVM for the ZF3-ZF4 array (top), experimental (middle) and predicted by expanded SVM for the ZF1-ZF2 array (bottom).

Tramtrack is a developmental protein (38) with eight known isoforms (39) that can be subdivided into two groups whose members have identical C2H2-ZF compositions. Each of these isoform groups has two C2H2-ZF domains, and their specificities are listed in the FlyFactorSurvey database as ttk-PF and ttk-PA. Predictions using the polynomial SVM for each of the isoforms clearly distinguish between the two isoforms (Figure 5A). The ttk-PF isoform is well predicted with six out of seven positions correctly predicted, whereas the ttk-PA isoform shows an overlap over seven positions, of which four are considered correct.

Lola, another developmentally important protein in *D. melanogaster* (40), has 25 identified transcripts and DNA-binding specificities have been reported for 13 isoforms with distinct C2H2-ZF compositions (41). Each of these isoforms has two C2H2-ZF domains. We predict PWMs using the expanded SVM and find statistically significant matches for 6 out of 13 isoforms (Supplementary Figure S9). Prediction quality for these isoforms varies, with some isoforms (e.g. PD, PG/PI, PL, PO, PT/PU and PW) clearly showing agreement in most positions.

PWM alignment can also be used to identify which C2H2-ZF domains within a multidomain zinc finger protein are mediating its known binding specificity. CTCF is a well-known insulator protein that binds DNA and blocks the interaction between enhancers and promoters, thereby preventing transcription. CTCF contains 11 zinc finger domains and has been shown to recognize a ∼15 bp-long DNA-binding motif (42). Alignment of this experimentally determined motif with the one predicted by the expanded SVM (Figure 5C) suggests that only predicted nucleotide positions 13–25 (which correspond to zinc fingers 4–7) coincide with the experimentally observed recognition motif (Figure 5B). This result is consistent with previous observations (43,44) that suggests that zinc fingers 4–7 are primarily responsible for the interaction with DNA.

Hermaphrodite (HER) is a transcription factor involved in sex determination in *D. melanogaster* that has four C2H2-ZF domains that are arranged in two arrays that each consist of two domains. The experimental PWM reported for HER is unusual for a C2H2-ZF, as it has a low IC region in the middle of it (31). PWMs predicted for both arrays using the expanded SVM are similar to those experimentally observed (Figure 5C). Interestingly, however, while the predicted ZF1-ZF2 array matches the experimental PWM directly, the prediction for the ZF3-ZF4 array best matches the complementary strand of the given PWM. Alternatively, a much weaker but still notable match for the ZF3-ZF4 array is obtained in the same region without reverse complementing. In either case, we propose that the unusual DNA-binding specificity experimentally observed for HER reflects the architecture of C2H2-ZF domains in this protein: four fingers interacting together with DNA in two paired arrays.

Overall, these examples show that our approach is a promising way to identify which C2H2-ZF domains are responsible for DNA-binding function when DNA sequence motifs are known but structural data are unavailable.

## DISCUSSION

Because C2H2-ZFs are the most prevalent DNA-binding domain in metazoans, high accuracy predictions of their DNA-binding specificities would be a great aid in expanding our knowledge of regulatory networks. Further, due to their ability to bind a wide-range of DNA targets, they have proven to be a powerful platform for genome engineering (45,46), and tools for predicting their DNA-binding specificities can be leveraged for designing proteins to bind specific DNA regions amenable to targeting (47). Previous approaches, both bioinformatics (10–13) and molecular mechanics based (15,48–51), have had limited testing, primarily on the Zif268 model system and a small number of natural C2H2-ZF proteins. Our goal in this work is to predict DNA-binding preferences directly from protein sequence, and to systematically assess how well C2H2-ZF specificity can be predicted for naturally occurring proteins.

We have shown that for ∼80% of the zinc fingers in our data set, we are able to predict PWMs that significantly match their known binding specificities. Further, we find that the expanded binding model has consistently better overall performance than the canonical model in predicting the specificities of natural C2H2-ZF proteins, and has similar performance to (and sometimes even outperforms) a more complex polynomial model that incorporates pairwise dependencies between all canonical contacts. This performance improvement supports our earlier finding that additional contacts beyond those in the canonical model are important for C2H2-ZF binding specificity, and can be used to better predict DNA-binding specificities (17). Though we have tested our approach for inferring PWMs using empirical energies derived via SVMs, we note that alternate scoring schemes may also be used, including those based on neural networks (13) or simple biophysical rules (52), both of which have already been applied towards predicting C2H2-ZF binding sites, or random forests and other classifiers that have been applied to predict the DNA-binding specificities for other classes of transcription factors (53) and may additionally be effective for C2H2-ZF domains.

Despite our progress, many challenges remain in predicting DNA-binding specificities at the protein level. In many known cases, only a subset of C2H2-ZF domains in a protein work together to bind DNA. Most of the well-studied C2H2-ZF arrays have nine residues between the last His residue and the first Cys residue of the next finger (as in the Zif268 protein). However, some C2H2-ZF arrays have longer linkers that also bind continuous DNA sequences. In this work, we have assumed that adjacent C2H2-ZF domains that are within 12 amino acids of each other bind DNA as a single array. However, C2H2-ZF domains that are further away from each other in the sequence may also work together. Further, when predicting binding specificities at the protein level, we do not know which of the observed ZF arrays are actually DNA binding; indeed, many C2H2-ZF domains are known to bind protein or RNA and may not bind DNA in a specific manner (54). As yet, no methods exist for predicting which C2H2-ZF domains bind DNA; for this reason, in our online tool, we allow the user to specify the C2H2-ZF domains for which binding specificities should be predicted.

Another complication is that consecutive C2H2-ZF domains that bind DNA together in a canonical fashion tend to do so with an overlapping nucleotide position. In our current work, we have predicted the nucleotide frequency of the overlap position using a weighted average of frequencies predicted using each of the fingers individually. However, it remains possible that longer range interactions, not considered in the protein–DNA interaction interface models studied here, can affect how adjacent C2H2-ZF domains cooperate with each other in binding DNA. For example, it has been observed that many C2H2-ZF proteins designed via modular assembly do not have their intended binding specificities (55); however, these modular assembly approaches have largely considered 3bp subsites, whereas we are explicitly taking the overlap position into account.

In order to uncover what types of PWMs are most difficult for our approaches to predict, we have also assessed performance based on column-wise nucleotide composition. We have found that A/T-rich targets are the most difficult for current methods to predict. This reflects the lack of A/T targets in our training data sets, and we believe that as more diverse examples of C2H2-ZF binding interfaces are uncovered, significant improvements in predicting C2H2-ZF binding specificities will result. We note however that while experimental PWMs are being rapidly determined for natural C2H2-ZF proteins as well as for other transcription factors via a variety of experimental techniques (8,56), in most of these cases, it is not known which C2H2-ZF domains interact with DNA or how the amino acids within these domains are contacting DNA. Thus, to date it has proved difficult to use these data to train models for predicting DNA-binding specificities. Even evaluating when a predicted binding specificity matches a known one has challenges, as it is necessary to align predicted and known PWMs. Though many approaches exist for aligning PWMs (57), there is not general agreement about which is the best to use and this introduces some uncertainty in the evaluation process. Nevertheless, we have shown in a few case studies that it is possible to use our approach for predicting PWMs along with PWM alignment to uncover which domains are responsible for observed experimental binding specificities. As more large-scale data are obtained about which fingers in C2H2-ZF proteins are interacting with DNA, this can be tested more systematically.

In sum, we have developed a general approach for estimating DNA-binding specificities for proteins containing C2H2-ZF domains, and have made this tool available online at http://zf.princeton.edu. We have quantitatively assessed the accuracy of our predictions on a diverse set of proteins for which PWMs have been experimentally determined, and have shown that our predicted PWMs significantly match experimental PWMs for over 80% of them. As new high-throughput experimental techniques are increasingly applied to quantitatively probe large numbers of C2H2-ZF protein-DNA binding interfaces, especially in designed systems where domain interactions are known (58–61), we expect that future models based on this data will yield even more accurate predictions of binding specificities.

## SUPPLEMENTARY DATA

Supplementary Data are available at NAR Online.

## FUNDING

This research was supported in part by NIH [R01 GM076275] and NSF [ABI 1062371]. Funding for open access charge: NIH [R01 GM076275].

## Supplementary Material

Supplementary Data
