# Supplementary material for: De novo prediction of DNA-binding specificities for Cys2His2 zinc finger proteins
Source: Nucleic Acids Res. 2013 Oct 3;42(1):97–108. doi: 10.1093/nar/gkt890 (PMC3874201; doi:10.1093/nar/gkt890)
Supplement: Supplementary Data [file supp_42_1_97__index.html]

De novo prediction of DNA-binding specificities for Cys2His2 zinc finger proteins — De novo prediction of DNA-binding specificities for Cys2His2 zinc finger proteins — Supplementary Data 

# De novo prediction of DNA-binding specificities for Cys2His2 zinc finger proteins

## Supplementary Data

files

**Files in this Data Supplement:**

- Supplementary Data - pdf file
