# Supplementary material for: De novo prediction of DNA-binding specificities for Cys2His2 zinc finger proteins
Source: Nucleic Acids Res. 2013 Oct 3;42(1):97–108. doi: 10.1093/nar/gkt890 (PMC3874201; doi:10.1093/nar/gkt890)

Supplementary materials

Figure S1. Examples of nucleotide alignments and their PCC and IC-weighted PCC scores.

logos: predicted experi-mental	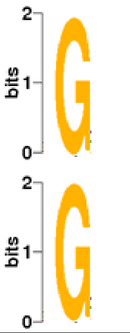	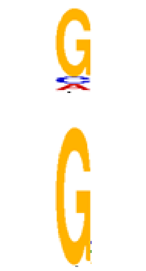	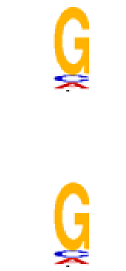	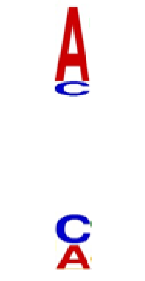	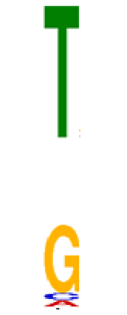	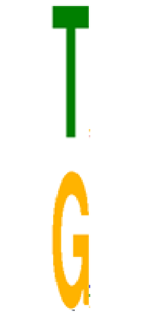	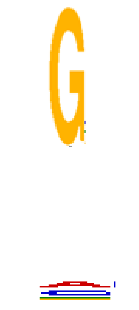	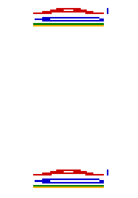	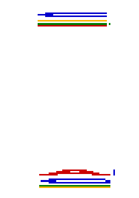
type of alignment	perfect	good agreement	lower IC	weak agreement	lower IC disagree-ment	disagree-ment	certain on uncertain	uncertain same order	uncertain different order
PCC	+1.000	+1.000	+1.000	+0.625	-0.226	-0.333	-0.309	+1.000	-0.857
IC-weighted PCC	+1.000	+1.000	+0.500	+0.250	-0.113	-0.333	0.000	0.000	0.000
result	considered correct with 0.25 threshold				not considered correct with 0.25 threshold				

Figure S2. Distributions of perfect match (blue) and random mismatch (green) IC-weighted PCC scores. Nucleotide columns were randomly selected from experimental PWMs in the Jaspar database. Each blue dot corresponds to the IC-weighted PCC score of a random column against itself, and is plotted as a function of the IC of the column. To generate the distribution of mismatch scores, for each random column c1 selected from JASPAR, we randomly selected an additional 100 columns from JASPAR where the most frequent nucleotide of these columns differed from that of c1. We then plot the mean score of c1 against these columns, along with the standard deviation, in green.

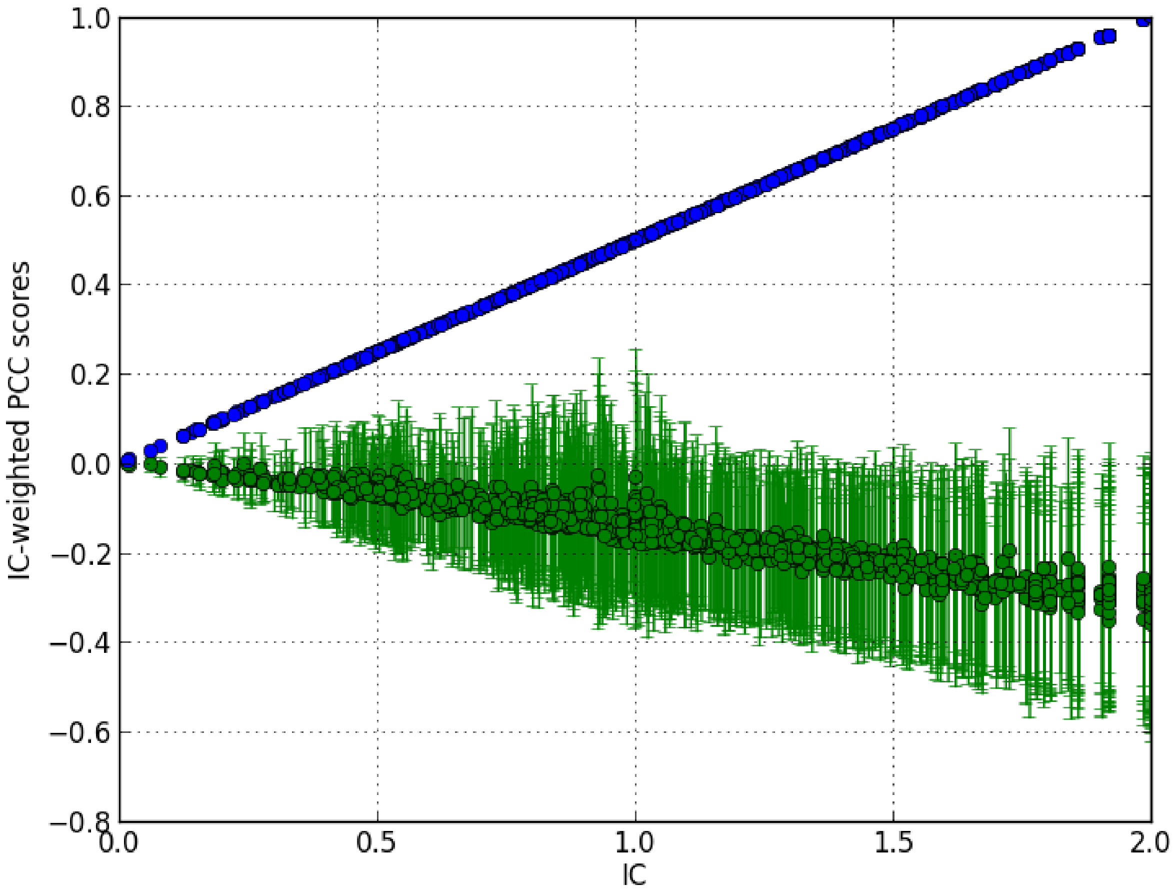

Figure S3. Performance of canonical (red), expanded (blue) and polynomial (black) SVMs in predicting natural C2H2-ZF proteins in the Jaspar data set. (A) Distribution of IC-weighted PCC scores in aligned PWMs. For each threshold of IC-weighted PCC score (x-axis), we plot the fraction of columns in trimmed experimental PWMs that achieve a score that high in their alignments to predicted PWMs. (B) Fraction of correctly predicted positions in PWMs. We give the percent of proteins (y-axis) whose PWMs have at least a given percent of correct columns (x-axis), using an IC-weighted PCC threshold of 0.25 to determine whether an aligned column is correct. (C) The percent of correctly predicted proteins at various p-values.

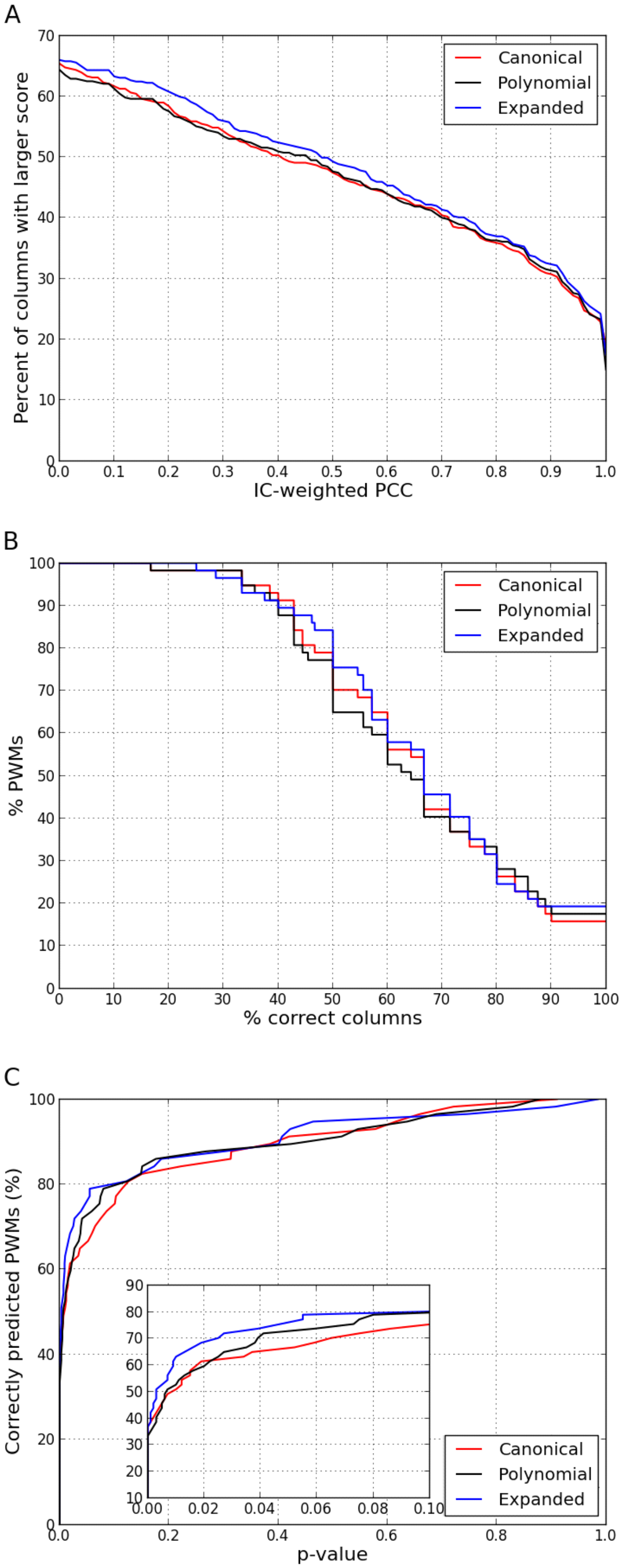

Figure S4. Performance of canonical (red), expanded (blue) and polynomial (black) SVMs in predicting natural C2H2-ZF proteins in the human data set. (A) Distribution of IC-weighted PCC scores in aligned PWMs. For each threshold of IC-weighted PCC score (x-axis), we plot the fraction of columns in trimmed experimental PWMs that achieve a score that high in their alignments to predicted PWMs. (B) Fraction of correctly predicted positions in PWMs. We give the percent of proteins (y-axis) whose PWMs have at least a given percent of correct columns (x-axis), using an IC-weighted PCC threshold of 0.25 to determine whether an aligned column is correct. (C) The percent of correctly predicted proteins at various p-values.

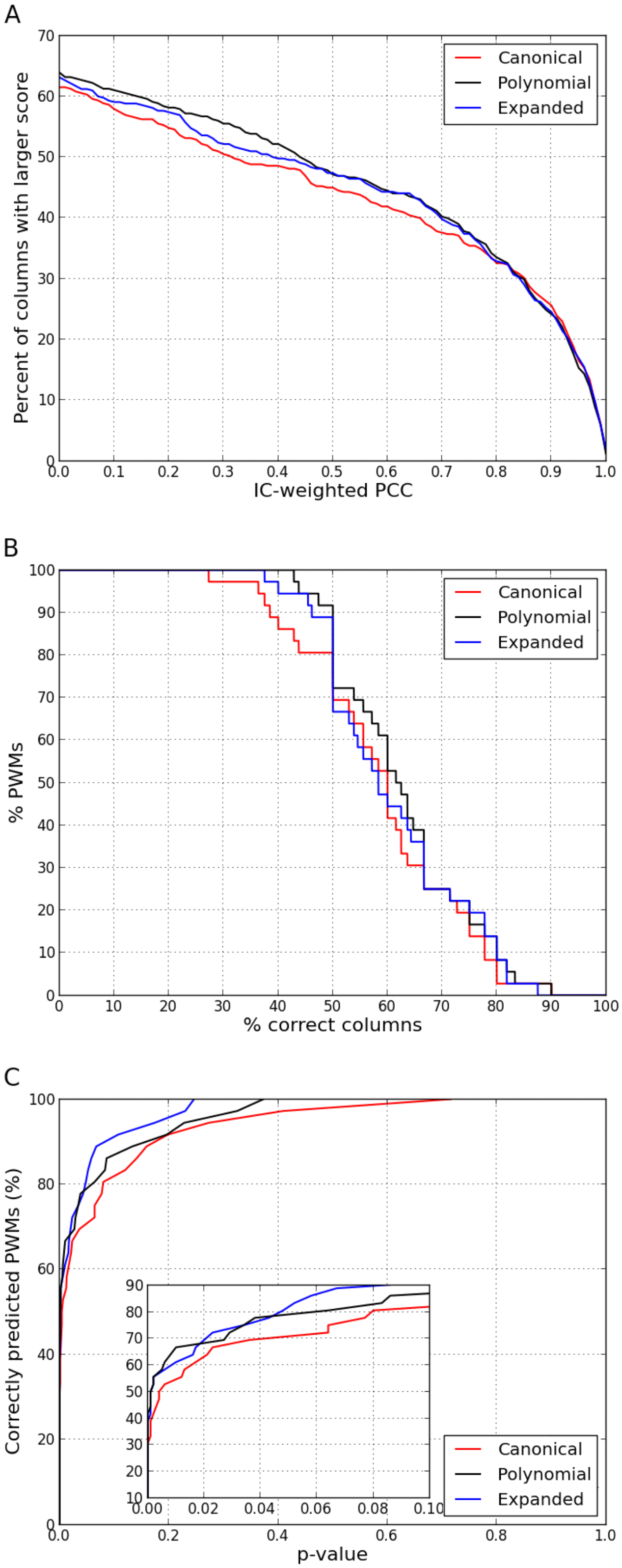

Figure S5. Performance of canonical (red), expanded (blue) and polynomial (black) SVMs in predicting natural C2H2-ZF proteins in the yeast data set. (A) Distribution of IC-weighted PCC scores in aligned PWMs. For each threshold of IC-weighted PCC score (x-axis), we plot the fraction of columns in trimmed experimental PWMs that achieve a score that high in their alignments to predicted PWMs. (B) Fractions of correctly predicted positions in PWMs. We give the percent of proteins (y-axis) whose PWMs have at least a given percent of correct columns (x-axis), using an IC-weighted PCC threshold of 0.25 to determine whether an aligned column is correct. (C) The percent of correctly predicted proteins at various p-values.

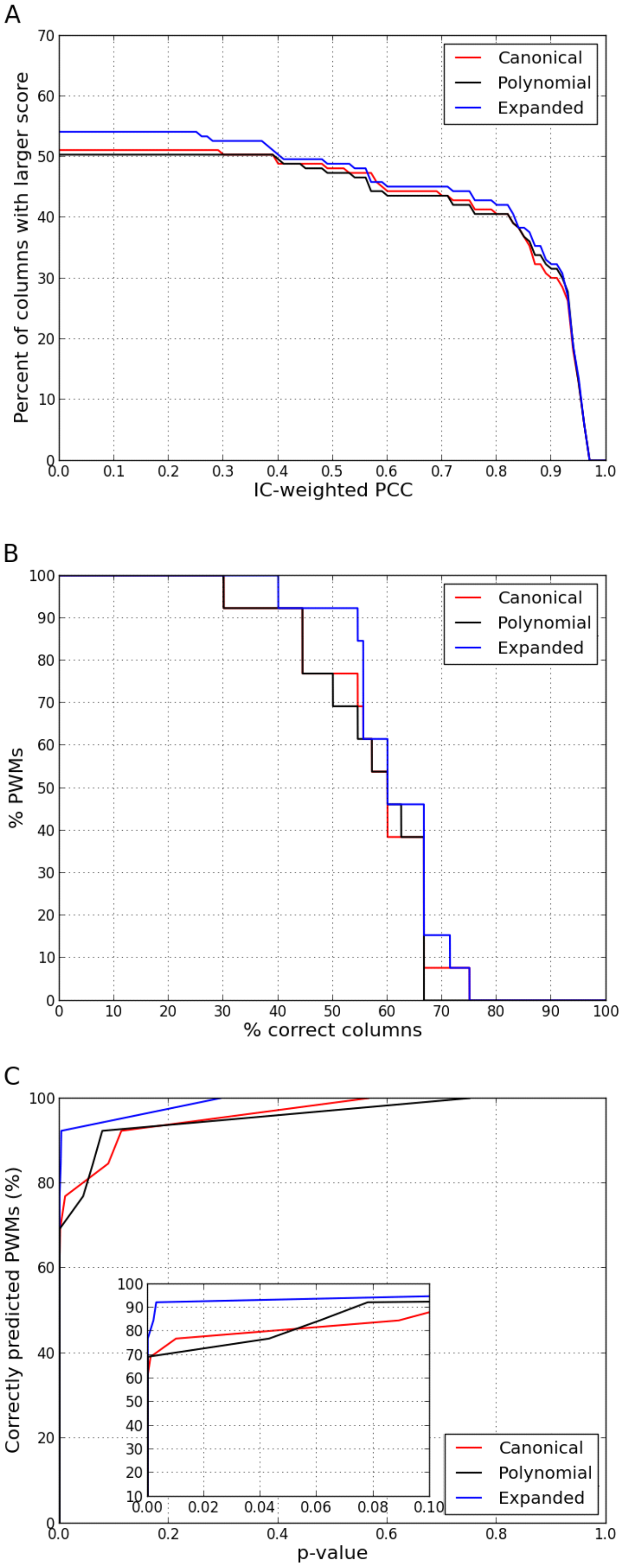

Figure S6. Performance of canonical (red), expanded (blue) and polynomial (black) SVMs in predicting natural C2H2-ZF proteins in the mouse data set. (A) Distribution of IC-weighted PCC scores in aligned PWMs. For each threshold of IC-weighted PCC score (x-axis), we plot the fraction of columns in trimmed experimental PWMs that achieve a score that high in their alignments to predicted PWMs. (B) Fractions of correctly predicted positions in PWMs. We give the percent of proteins (y-axis) whose PWMs have at least a given percent of correct columns (x-axis), using an IC-weighted PCC threshold of 0.25 to determine whether an aligned column is correct. (C) The percent of correctly predicted proteins at various p-values.

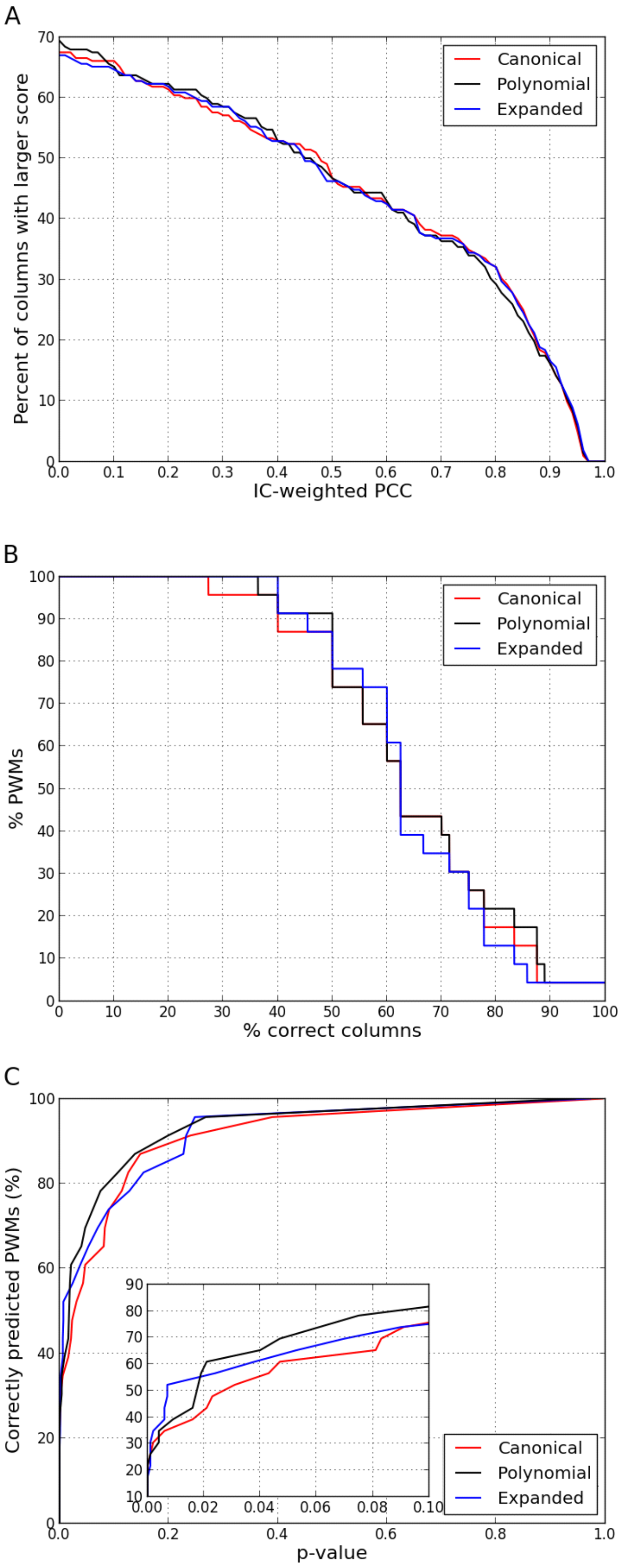

Figure S7. Performance of canonical (red), expanded (blue) and polynomial (black) SVMs in predicting natural C2H2-ZF proteins in the fly data set. (A) Distribution of IC-weighted PCC scores in aligned PWMs. For each threshold of IC-weighted PCC score (x-axis), we plot the fraction of columns in trimmed experimental PWMs that achieve a score that high in their alignments to predicted PWMs. (B) Fractions of correctly predicted positions in PWMs. We give the percent of proteins (y-axis) whose PWMs have at least a given percent of correct columns (x-axis), using an IC-weighted PCC threshold of 0.25 to determine whether an aligned column is correct. (C) The percent of correctly predicted proteins at various p-values.

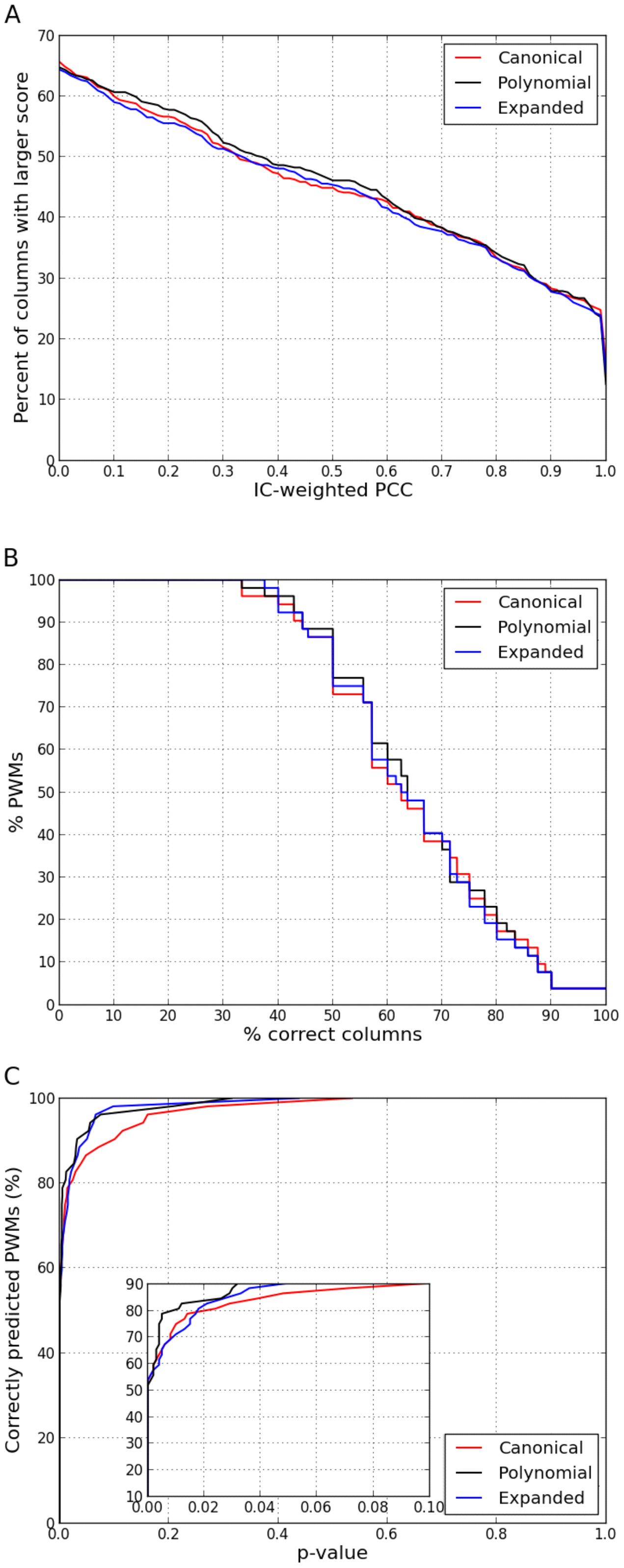

Figure S8. The percent of correctly predicted proteins for the combined data set at various p-values by the SVM methods and KFM05, the method based on the Kaplan at al. (2005) PLoS Comput Biol. 1(1):e1. For KFM05, we used their published probability distributions, and handled arrays of C2H2-ZF domains and the overlap nucleotide position as was done for the SVM methods.

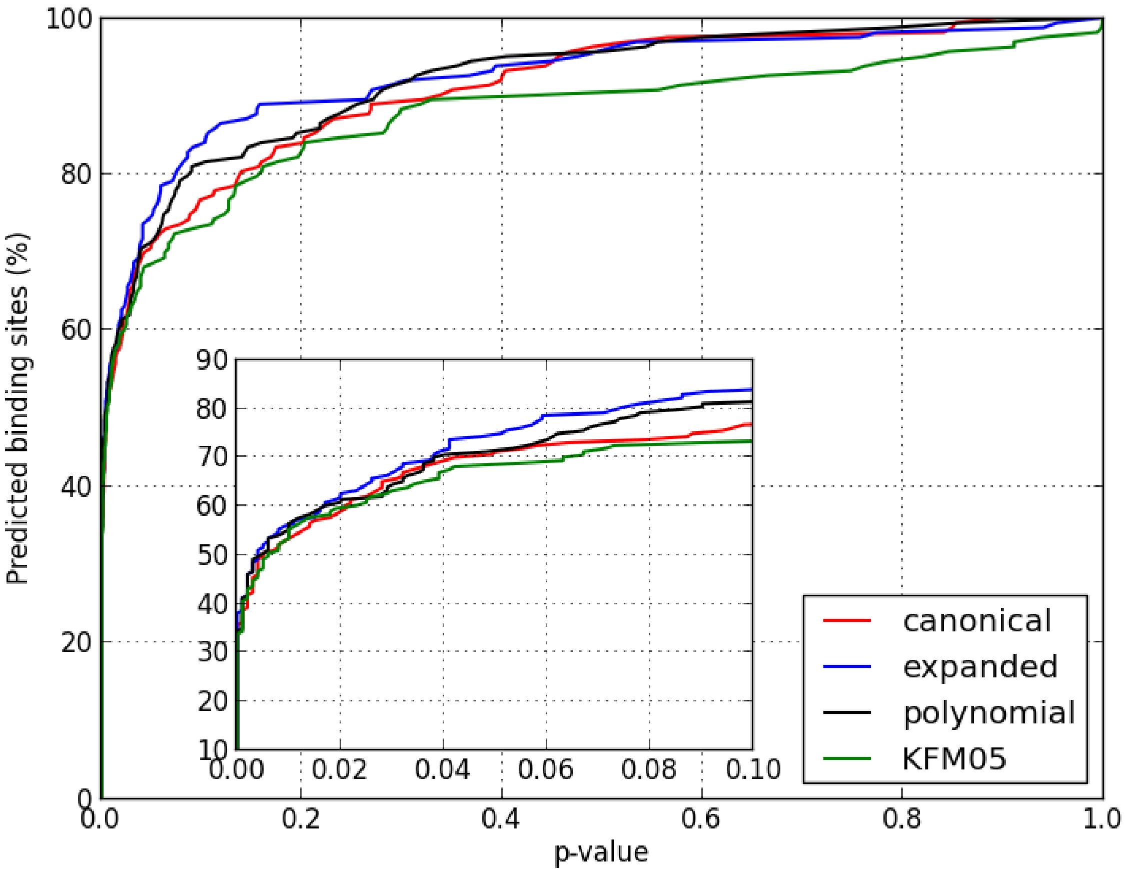

Figure S9. Experimentally determined PWMs (top) and PWMs predicted by the expanded SVM model (bottom) for various lola protein isoforms.

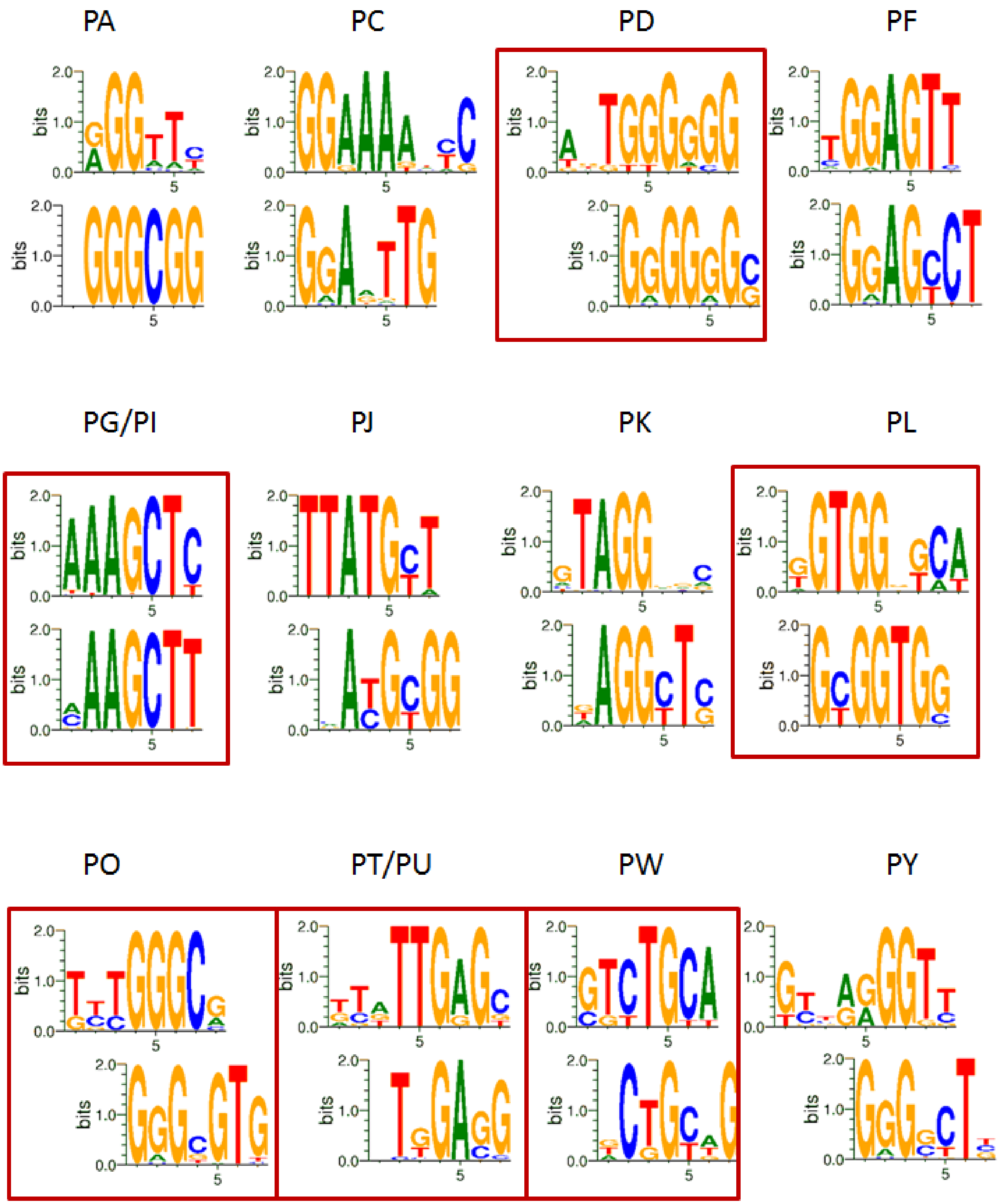

Supplement: Supplementary Data [file supp_gkt890_nar-02194-h-2013-File002.pdf]
